# Agreement of Bioreactance Cardiac Output Monitoring With Thermodilution in Healthy Standing Horses

**DOI:** 10.3389/fvets.2021.701339

**Published:** 2021-08-03

**Authors:** Klaus Hopster, Samuel D. A. Hurcombe

**Affiliations:** Department of Clinical Studies, School of Veterinary Medicine, University of Pennsylvania, Philadelphia, PA, United States

**Keywords:** hemodynamic, equine, bioimpedance, flow, dobutamine

## Abstract

Bioreactance is the continuous analysis of transthoracic voltage variation in response to an applied high frequency transthoracic current and was recently introduced for non-invasive cardiac output measurement (NICOM). We evaluated NICOM compared to thermodilution (TD) in adult horses. Six healthy horses were used for this prospective, blinded, experimental study. Cardiac output (CO) measurements were performed simultaneously using TD and the bioreactance method. Different cardiac output scenarios were established using xylazine (0.5 mg/kg IV) and dobutamine (1.5–3 mcg/kg/min). Statistical analysis was performed by calculating the concordance rate, performing a regression analysis, Pearson correlation, and Bland Altman. The TD-based CO and NICOM values were highly correlated for low, normal and high CO values with an overall correlation coefficient. A 4-quadrant plot showed an 89% rate of concordance. The linear regression calculated a relationship between NICOM and TDCO of Y = 0.4874 · X + 0.5936. For the corrected Bland Altman agreement, the mean bias and lower/upper limits of agreement were −0.26 and −3.88 to 3.41 L/min, respectively. Compared to TD, bioreactance- based NICOM showed good accuracy at induced low, normal, and high CO states in normal horses. Future studies performed under more clinical conditions will show if this monitor can help to assess hemodynamic status and guide therapy in horses in ICU settings and under general anesthesia.

## Introduction

Measurement of cardiac output (CO) and, sometimes more importantly, changes in CO can be extremely useful when assessing circulatory function. An easy, fast and reliable method to measure the CO is not only required for research purposes but also for clinical settings and currently missing in equine medicine.

Thermodilution technique as a measure of cardiac output (CO) was first introduced in the 1950's and is since then widely used in all kinds of species and recognized as a gold standard for evaluating and determination of the CO (1, 2). Notably in the clinical setting, however, it is considered being relatively invasive due to the necessity of placing intracardiac and -pulmonary artery catheters and therefore often not utilized in equine patients (3). As a result, there is a continuing search for a method of CO measurement that is less invasive than its predecessors.

A number of non-invasive methods of assessing CO have been studied in horses in the past, with transesophageal or transthoracic Doppler echocardiography (4, 5), Lithium dilution techniques with and without pulse contour analysis (6, 7), and carbon dioxide breath analysis currently available (8, 9). In human medicine thoracic bioimpedance was one of the first and currently most widely used non-invasive method for CO monitoring due to its easy application and non-invasiveness (10, 11).

Bioelectrical impedance analysis or bioimpedance is a widely used method for estimating fluid shifts in a biological system. In bioimpedance, a weak electric current flow through the body and the voltage is measured in order to calculate impedance (resistance) of the body (12). Thoracic electrical bioimpedance uses change in impedance by an alternating current applied across the thorax to determine various hemodynamic parameters, including stroke volume, cardiac output, and thoracic fluid content (12). Since the advent of the first available devices in the mid-1980's the method has become popular due to its ease of use (13). However, disadvantages of this non-invasive methodology are its inaccuracy impacted by electric noise and movement artifacts as well as its dependency of particular and accurate electrode placement (14).

To minimize the error rate and improve accuracy and reliability the bioimpedance methodology underwent further modification. While bioimpedance analyses only on the measured changes in signal amplitude the newly introduced bioreactance analyses the time delay or relative phase shift of the oscillating currents making the signal and analysis more reliable and less prone to motion artifacts and positioning errors (15–17). When compared to thermodilution the bioreactance methodology showed good agreement in swine and humans (15, 16). Further, validation studies of this technology in human and dogs have shown good correlation and agreement of bioreactance values compared with CO values obtained using other devices (16, 18–20) while other studies have reported a large limit of agreement and high percentage errors of up to 80% (21, 22). Validation studies for equine patients are lacking.

The goal of this study was to evaluate the performance of a bioreactance device for cardiac output measurements in adult horses. We hypothesized that there would be good agreement between TD CO measurements and bioreactance CO measurements across a range of CO states but that due to the anatomical differences between humans and horses a correction factor would be necessary to obtain valid values.

## Materials and Methods

### Study Design

This study was performed as a prospective, blinded, experimental study. The protocol was approved by the University's Institutional Animal and Use Committee (#807004) and six university-owned research/teaching horses (five mares and one gelding) were used. These six horses were also enrolled in another separate experimental study on cardiovascular performance. An a priori power analysis (alpha 0.05 and beta 0.8) revealed that minimum five horses would be necessary to detect a clinically relevant agreement; a sixth horse was included to account for possible loss of data.

Horses were between 6 and 15 years old and body weight ranged from 420 to 580 kg. They were free of cardiovascular and respiratory disease based on physical examination, packed cell volume, and total solids values.

### Thermodilution Cardiac Output Measurement

Before the experiment the skin over both jugular veins was clipped and aseptically prepared for intravenous catheter placement. After infiltration of the skin with lidocaine (Lidocaine 2%, Henry Schein, Dublin, Ohio) a 12 G catheter was placed into the left jugular vein and two 8 Fr catheter introducers were separately placed in the right jugular vein to facilitate the placement of balloon tipped catheters. One Swan-Ganz standard thermodilution pulmonary artery catheter (Criticath™ 7 Fr/110 cm, BD Medical, Franklin Lakes, NJ) was placed into the main pulmonary artery and a second Swan-Ganz catheter was placed into the right atrium. Correct placement was confirmed by visual inspection of the pressure waveforms (23).

For the measurement of the cardiac output (CO) by thermodilution (TD), iced saline solution (1 ml/15 kg bwt) was injected manually through the catheter into the right atrium at end-expiration over a time period of ~6 s. The temperature of the injectate was measured *via* an inline temperature probe and the temperature change in the pulmonary artery was analyzed to calculate the CO (Cardiocap/5, Datex-Ohmeda Inc, Madison, WI). Five injections were performed over a time of 5 min and the average of the closest three CO values was used.

### Bioreactance Cardiac Output Measurements

The bioreactance-based non-invasive CO measurement system is based on an analysis of relative phase shifts of an oscillating current that occur when these current traverses the thoracic cavity and is described in detail at Keren et al. (15). The used non-invasive CO measurement system (NICOM, Cheetah Medical, Inc., Portland, OR) uses a high-frequency (75 kHz) sine wave generator and four dual-electrode “stickers” that are used to establish electrical contact with the body.

During initial pilot work different sensor placement sites and pairings were compared to determine the location and orientation used below that provided most consistent measurements and resulted in detection of a continuous ECG and NICOM waveforms. During this pilot work the sensors were either placed in a horizontal orientation with one sensor over the shoulder joint and the second sensor at the same height over the 12th intercostal space or in a vertical orientation with one sensor under the withers and one sensor at the level of the elbow. Repeated measurements showed that the vertical orientation resulted in less artifacts and more consistent measurement results.

**Figure 1:**
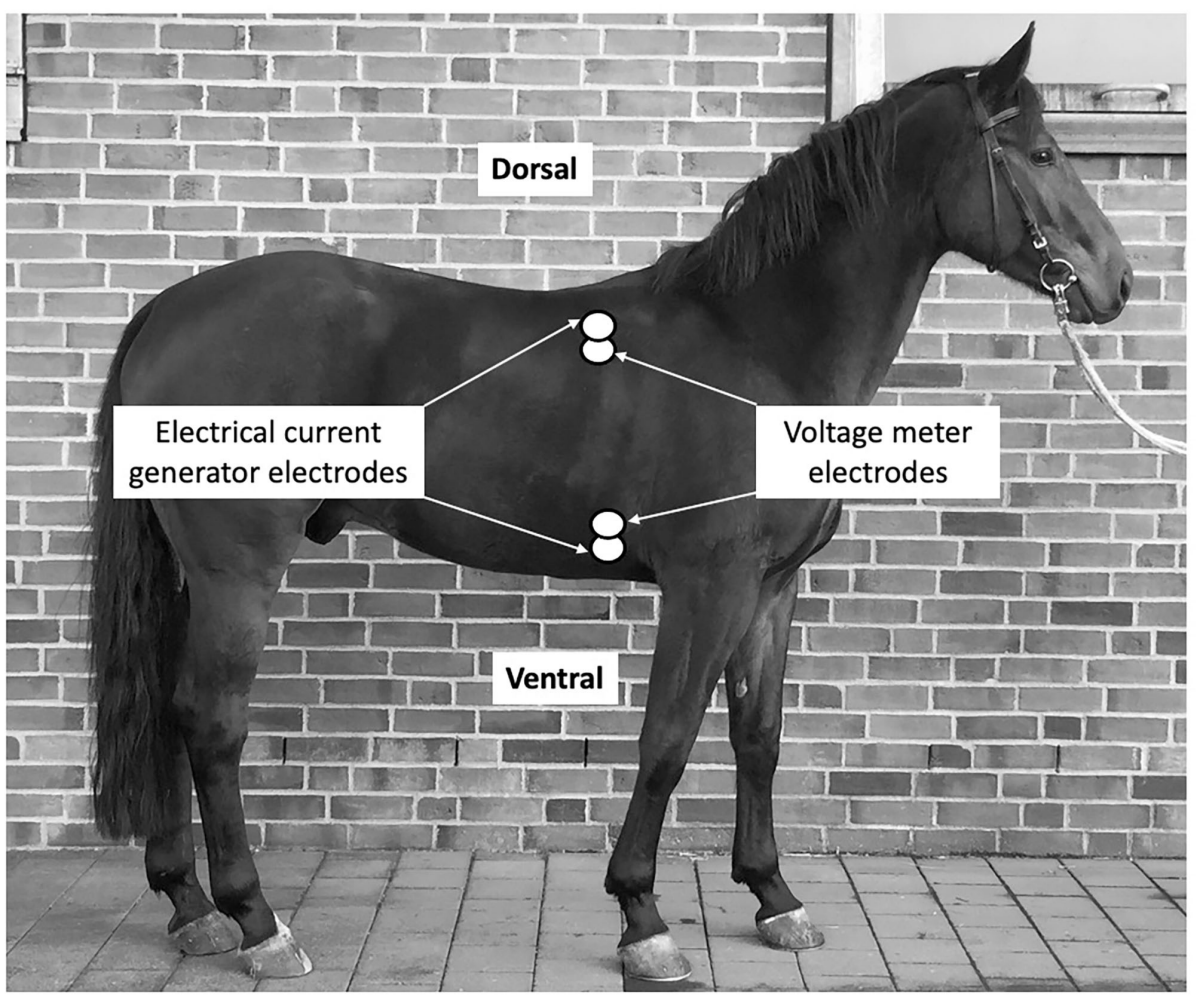
Placement of the sensor stickers on the right side of the thorax of the horse. Sensors are indicated be white double-circles.

Therefore, for this study the bioreactance sensors were placed on the thoracic locations as pairs; two on the left and two on the right side, one sensor just below the withers (dorsal sensor), and another sensor at the level of the elbow (ventral sensor). The sites for sensor placement were clipped and shaved before being wiped off with isopropyl alcohol and allowed to air dry. The sensors were oriented vertically such that the generator was above its corresponding amplifier for the dorsally placed sensor and below its corresponding amplifier for the ventrally placed sensor. The dorsal sensors were placed ~15 cm below the withers in the 8th intercostal space. The ventral sensors were in line with the dorsal sensors ~15 cm above the level of the sternum (Figure 1).

Within each sticker, one electrode is used by the high-frequency current generator to inject the high-frequency sine wave into the body, while the other electrode is used by the voltage input amplifier. As the stickers on a given side of the body are paired the currents are passed between the outer electrodes of the pair and voltages are recorded from between the inner electrodes. A non-invasive CO measurement signal is determined separately from each side of the body, and the final non-invasive CO measurement signal is obtained by averaging these two signals.

The system's signal processing unit detects the relative phase shift of the input signal relative to the injected signal. The phase shift between the injected current and output signal received from the thorax is due to presumed changes in blood volume in the aorta.

### Experimental Design

Horses were placed in stocks and simultaneous thermodilution (TDCO) and bioreactance CO (NICOM) measurements were performed for a total of 5 h. The evaluator responsible for the NICOM measurements (SDH) was unaware measurements obtained by TDCO. Order of measurements was always the same with TDCO measurements performed first and the NICOM value immediately following completion of TDCO being recorded and used for analysis.

During the first 2 h horses were left undisturbed in stocks and CO measurements were performed every 15 min. Thereafter, horses were sedated with xylazine 0.5 mg/kg (X-ject®, Henry Schein, Dublin, Ohio, USA) intravenously to induce low cardiac output states and measurements were continued every 10 min for another hour.

After a 2-h break horses received a constant rate infusion of dobutamine (Dobutamine®, Hospira Inc, Lake Forest, Illinois, USA), 1.5 mcg/kg/min for 30 min and 3 mcg/kg/min for 30 min each, to induce high cardiac output states. During the dobutamine infusion paired CO measurements were performed every 10 min followed by another hour of undisturbed measurements every 15 min.

This design resulted in a total of 26 paired CO measurements per horse, six paired measurements for low, 14 paired measurements for “normal” and six paired measurements for high CO situations. During the study period horses were housed in stalls including daily turnout in on a small paddock with free access to hay and water. Horses were monitored for adverse events throughout and for 48 h after completion of the experiment and then transferred back to the hospital teaching herd.

### Statistical Analysis

The approach to modeling the relationship between thermodilution and bioreactance a multi-level modeled linear regression analysis was performed with TDCO values considered to be the explanatory variables and NICOM values considered to be dependent variables.

Data were assessed for normality based on examination of a histogram and normal plot of the residuals. For most analysis cardiac output values were grouped into three categories: LOW, NORMAL, and HIGH. Values measured after xylazine administration for up to 1 h were considered LOW, all baseline measured values were considered NORMAL and values obtained during dobutamine administration were grouped into the HIGH category.

Pearson correlation coefficient was calculated to assess the correlation between TDCO and NICOM (high, low, and normal CO) for all phases together.

To evaluate and compare the repeatability of TD and NICOM, the mean percentage error (MPE) of the repeated measurements of baseline evaluations before xylazine and dobutamine administration was calculated.

Agreement between regression- corrected NICOM and TDCO was quantified using the Bland Altman method for repeated measurements for the low, normal and high cardiac output values (24). The bias was reported as the mean difference between NICOM-corrected and TDCO. A positive bias reflected underestimation and a negative bias reflected overestimation of TDCO by NICOM. The limits of agreement were calculated as bias ± (1.96 × standard deviation of the bias).

Concordance rate was calculated. The values lying in the upper right and lower left zone indicated the paired differences in cardiac output values with the same direction of change (25). A central zone data with difference in cardiac output values of lesser than 1 L were considered noise and excluded from analysis.

All analyses were done with a commercial statistical software (GraphPad Prism v8. LaJolla, CA) and significance was set at *P* < 0.05.

## Results

A total of 156 sets of paired NICOM and TDCO measurements were obtained and compared. No significant change in heart rate or respiratory rate was appreciated throughout the experiment.

The repeatability of the NICOM measurements was satisfactory with a MPE of 8.7% for repeated baseline measurements.

**Figure 2:**
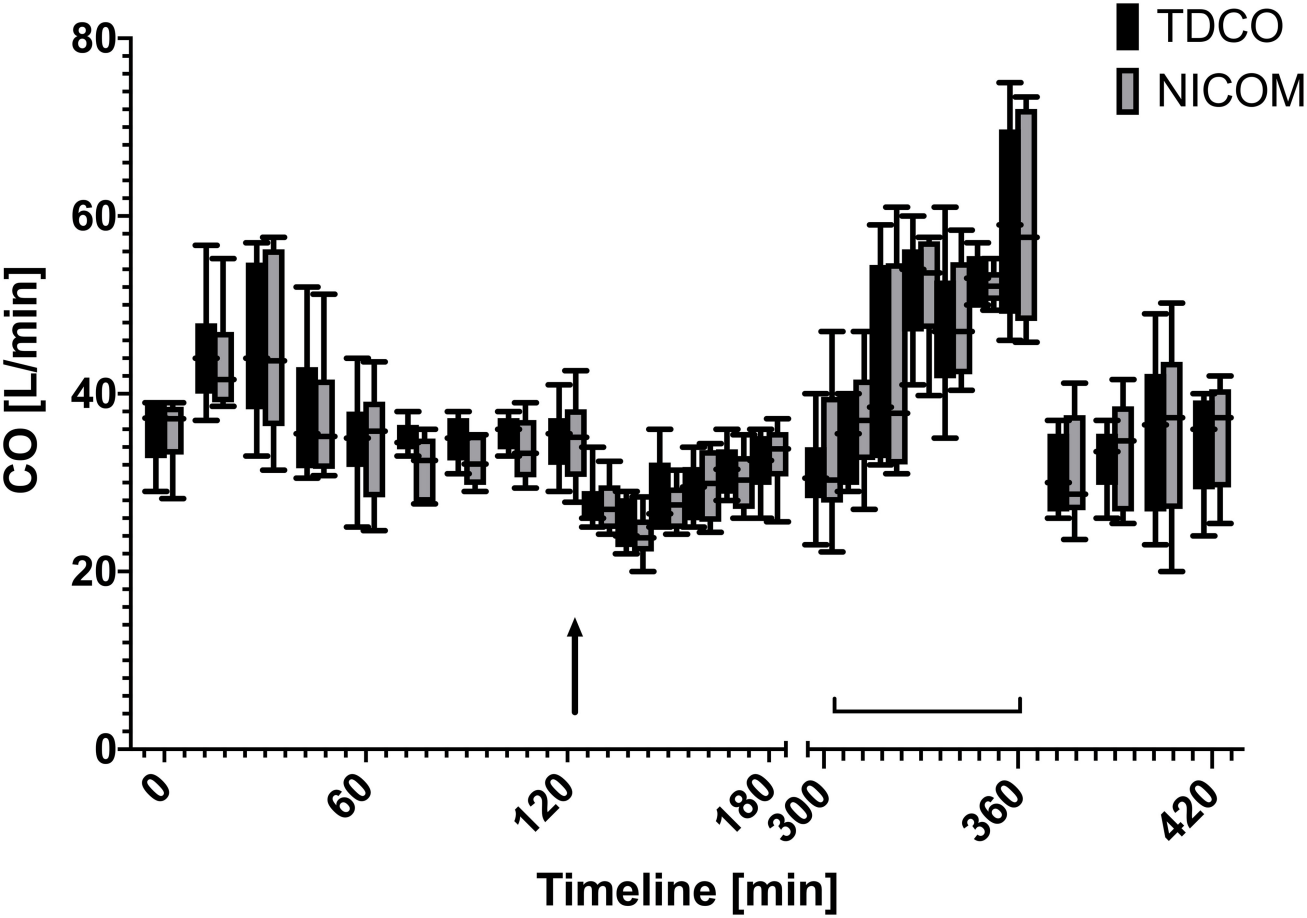
Box and whiskers plot of cardiac output values over time measured using thermodilution (TDCO) and corrected bioreactance (NICOM) values (×1.962) at different time points during rest, after xylazine bolus (125 min, black arrow), and during dobutamine CRI (305–360 min, brackets).

The administered drugs xylazine and dobutamine resulted in a CO range from 22 to 75 L/min for TD and CO range from 10.2 to 37.4 L/min for uncorrected NICOM over time during baseline measurements, after xylazine bolus and during dobutamine CRI (Figure 2).

The trend analysis for NICOM and TDCO revealed a concordance of 89% with significant correlation (*r*^2^ = 0.9, *p* < 0.001) (exclusion zone <1 L/min).

The linear regression analysis performed for all data points at all CO situations calculated a relationship between NICOM and TDCO of Y = 0.4874 · X + 0.5936 with a goodness of fit of *r* = 0.96 and 95% confidence intervals for the slope from 0.4743 to 0.5006. For the low CO measurements, a factor of 0.496 and for the high CO measurements a factor of 0.437 was determined.

**Figure 3:**
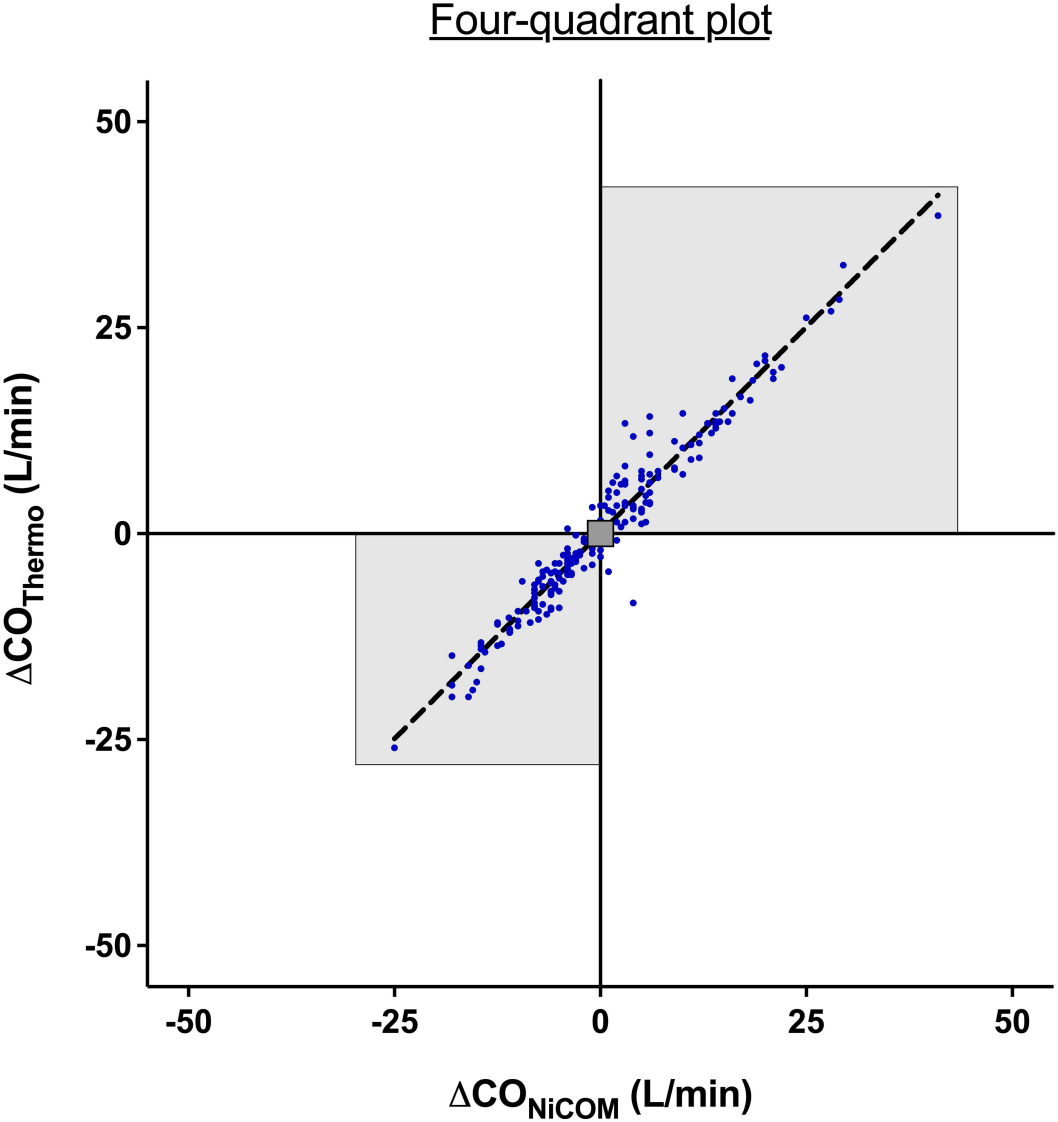
Four-quadrant plot to assess the agreement in the direction of change between bioreactance and thermodilution. An exclusion zone of values <1 L/min was applied. The highlighted area represents the two quadrants containing the delta cardiac output pairs with the same direction of change. The dotted line represents the line of identity where x = y.

The four-quadrant plot describing the agreement between two methods and the change in CO is shown in Figure 3.

The NICOM and TDCO measurements showed a significant correlation for low, “normal” and high values (*p* < 0.001) with a correlation coefficient of *r*^2^ = 0.77 for low, *r*^2^ = 0.93 for “normal,” and *r*^2^ = 0.89 for high CO measurements.

**Figure 4:**
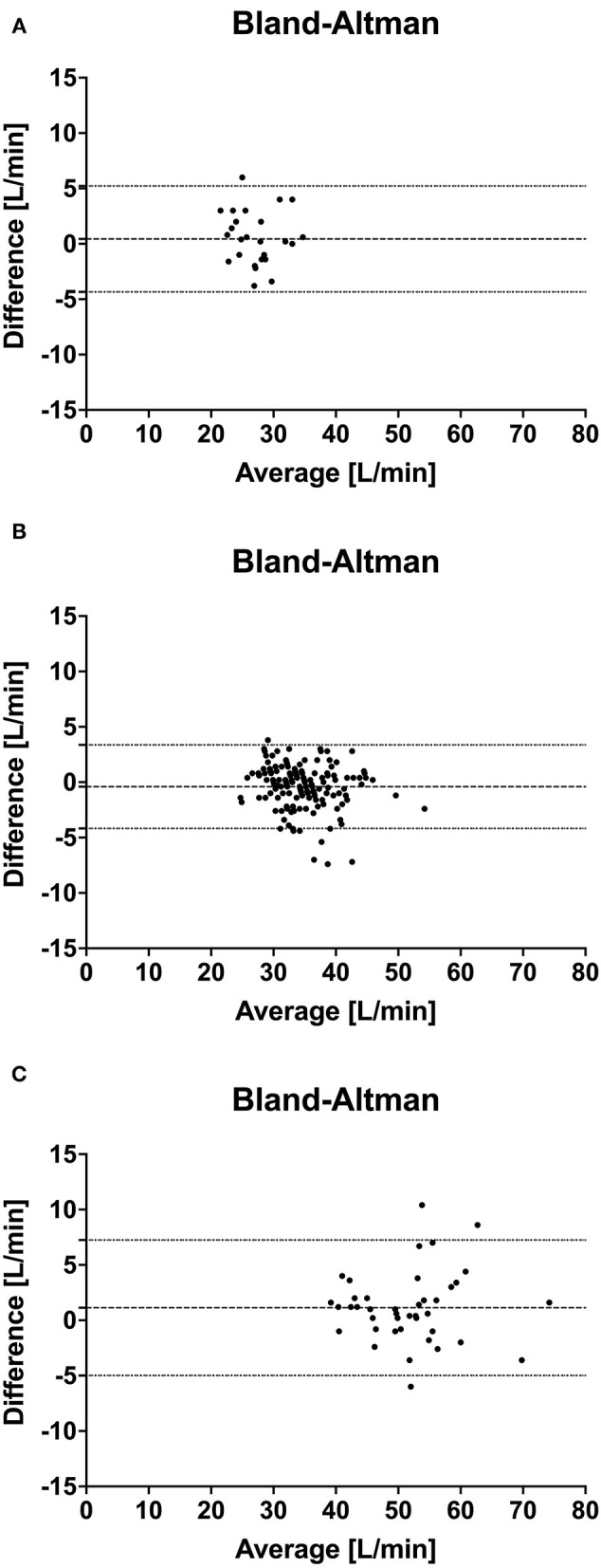
Bland Altman analysis of simultaneous cardiac output measurements using thermodilution (TDCO) and bioreactance (NICOM) in 6 healthy horses during xylazine induced low CO **(A)**, baseline normal CO **(B)** and dobutamine induced high CO **(C)** stages. The y-coordinate is the difference of the mean of each pair, and the x-coordinate is the mean of each pair. Dashed line marks the bias, the dotted line marks the limits of agreement.

For the Bland Altman agreement the NICOM values were corrected by the factor 1:0.4874 (= 2.05) determined by the regression analysis for all measurements. The mean bias, lower, and upper limit of agreement were 0.44 L/min and −4.34 to 5.2 L/min for low, −0.41 L/min and −4.18 to 3.36 L/min for normal and 1.13 L/min and −4.98 to 7.25 L/min for high CO measurements, respectively. Bland and Altman plots are shown in Figure 4.

No adverse events occurred in association with either NICOM or TDCO measurements in any horse at any time.

## Discussion

The bioreactance cardiac output measurements is a relatively new way for continuous and non-invasive measurement of CO that has been validated for accuracy in different species (15, 16, 18–20, 26). The present study involved simultaneous bioreactance and TDCO measurements and was designed to determine whether the continuous data provided by the bioreactance would be able to accurately measure CO in horses and further quantify changes or detect trends.

Thermodilution technique was used as gold standard measurement to evaluate accuracy of the NICOM. While pulmonary artery catheterization and thermodilution measurement of CO has been widely used in experimental settings, the routine use of TDCO determination in clinical equine practice has been limited due to its invasiveness and necessity for extensive equipment such as the CO computer and a high-speed injector to ensure that the large volumes of thermal indicator are delivered consistently (3).

Our data indicate that bioreactance measurements and the TDCO measurements show a strong correlation, and the linear regression analysis as well as concordance calculation indicates that bioreactance measurements needed to be corrected by the factor 2 (1:0.4874). The reason for the almost 50% underestimation of the NICOM could be the difference in cardio-vascular anatomy and physiology between human and equine patients as the used monitoring system is based on an algorithm developed for human patients. A recent study developed a 1D computer model for the equine arterial circulation comprising all major vessels of the arterial tree indicating significant differences in arterial pressure waves and wave forms as well as vessel performance for the horse (27). Furthermore, the significant differences in anatomy and physiology of the vascular structures between horses and humans and differences and cardiac performance such as stroke index and heart rate (28, 29) make the translation of inbuilt algorithms difficult. Therefore, it is not surprising that a correction factor was necessary to obtain accurate values. Another important factor to consider is the difference in cardiothoracic anatomical structure of human and horse (bipedal vs. quadrupedal) as well as the resultant difference in location of sensor placement. While the proportionality constant in the used NICOM device is derived from characteristics of humans (15) such equine based calibration constant is unknown. More studies with horses of different sizes, weights, and ages are needed to determine if other correction factors are necessary or the factor determined in this study represents an adequate constant for all studies in horses. The very good accuracy and performance of the NICOM, despite being described in the literature (16, 18–20), was not expected in that degree by the authors. However, despite the small bias we appreciated a wider LAO, particularly when higher CO states were tested. We can only speculate that the unique cardiovascular anatomy of the horse with a relatively large heart placed in the center of the chest contributes to the good performance of the NICOM. Further studies using different cardiovascular conditions are needed to confirm our findings.

For clinical application the detection of a trend in CO changes is of great importance as the measurement of actual values. Therefore, the second goal of this study was to evaluate whether the NICOM would detect changes in CO and provide accurate trends. For sufficient evaluation a wide range of different cardiovascular stages with high and low cardiac output values was induced by drugs that have known effect on the cardiovascular system and the cardiac output. Xylazine at comparable dosages as used in this study has shown to decrease the cardiac output by ~50% (30) and thermodilution as well as bioreactance measurements showed expected decreases in CO values for up to 35 min after xylazine bolus administration. To induce high cardiac output states dobutamine was administered at dosages of 1.5 and 3 mcg/kg/min. In anesthetized and in standing horses dobutamine infusion rates of 0.5 to 4 mcg/kg/min resulted in an increase of cardiac output up to 35% (31–33) which was also seen in our horses. These drug protocols resulted in CO values ranging from 22 to 75 L/min measured by TD and these trends and changes were detected by NICOM with great correlation (*r*^2^ = 0.978).

After correcting the values obtained by bioreactance using the regression factor 1.96, the Bland Altman analysis calculated a small bias for all three induced and investigated CO stages but a large LOA ranging from +5 to −7 L/min. Although, this LAO can still be considered acceptable being in the range of 15% it needs to be considered particularly in clinical settings in which decisions could be based on the measured CO values (24, 26). This also emphasized the importance of careful interpretation of these results obtained on a relatively small number of healthy horses.

No major adverse events were observed in the animals throughout the course of the study when a bioreactance system was used. This can be expected as the system is non-invasive.

### Limitations

The bioreactance methodology uses, like other non-invasive techniques, the mathematical concept of integration of the area under the flow pulse over time which assumes that the product of ventricular ejection and flow are proportional, and we used healthy and normovolemic horses for our investigations in which these concepts are giving. This can also explain the high correlation factor between thermodilution and bioreactance in our study which was higher than what is described in clinical studies investigating cardiovascularly compromised patients (15). Importantly, clinical patients may experience periods of low and non-linear flow in which the concept of proportionality of ejection and flow might not be valid (15). Therefore, our findings still need to be confirmed in the clinical setting and with horses experiencing cardiovascular compromise.

Another limitation is that the thermodilution technique was taken as a gold standard as it is the best available reference for CO monitoring in horses (1). Our interest was comparing the TDCO system with an automatic and non-invasive continuous monitoring tool. In this regard, the data showed that there is good correlation between TDCO - and bioreactance derived measurements of CO. However, thermodilution technique may not actually provide an accurate value for comparison and does not provide continuous measurements which further makes comparisons difficult.

## Conclusions

The current study shows that this device can perform well in standing, healthy horses.

The results of this study showed a good agreement of the bioreactance-based system for measurement of CO compared to thermodilution technique, but also a relatively wide limit of agreement (2SD). As a next step this methodology needs to be introduced into clinical settings and tested on a larger number of cardiovascularly compromised patients and on a larger range of age, size, and bodyweight to assess not only reliability of this technique but also performance under clinical conditions including practicability. The calibration factor we found in this study will need further evaluation in a larger and more heterogeneous dataset. Further, developing an algorithm which would account for patient-specific parameters will be necessary to improve clinical utility.

## Data Availability Statement

The original contributions presented in the study are included in the article/supplementary material, further inquiries can be directed to the corresponding author/s.

## Ethics Statement

The animal study was reviewed and approved by the protocol was approved by the University's Institutional Animal and Use Committee (#807004).

## Author Contributions

KH: study design, data collection, analysis and interpretation of data, and writing up of the first draft of the manuscript. SH: study design, data collection, analysis and interpretation of data, and critical revision of the manuscript. All authors contributed to the article and approved the submitted version.

## Conflict of Interest

The authors declare that the research was conducted in the absence of any commercial or financial relationships that could be construed as a potential conflict of interest.

## Publisher's Note

All claims expressed in this article are solely those of the authors and do not necessarily represent those of their affiliated organizations, or those of the publisher, the editors and the reviewers. Any product that may be evaluated in this article, or claim that may be made by its manufacturer, is not guaranteed or endorsed by the publisher.
